# ﻿The genus *Epeolus* Latreille, 1802 (Hymenoptera, Apidae) in Central Asia

**DOI:** 10.3897/zookeys.1181.110416

**Published:** 2023-10-06

**Authors:** Yulia V. Astafurova, Maxim Yu. Proshchalykin

**Affiliations:** 1 Zoological Institute, Russian Academy of Sciences, Saint Petersburg 199034, Russia Zoological Institute, Russian Academy of Sciences Saint Petersburg Russia; 2 Federal Scientific Center of the East Asia Terrestrial Biodiversity, Far East Branch of the Russian Academy of Sciences, Vladivostok 690022, Russia Federal Scientific Center of the East Asia Terrestrial Biodiversity, Far East Branch of the Russian Academy of Sciences Vladivostok Russia

**Keywords:** Anthophila, Apiformes, cleptoparasites, Palaearctic region, taxonomy

## Abstract

Available information about bees of the genus *Epeolus* in Central Asia is summarized. Twenty species are currently known from this area. Two new species are described: *E.albus* Astafurova & Proshchalykin, **sp. nov.** and *E.pesenkoi* Astafurova, **sp. nov.** Two species are newly recorded from Central Asia: *E.asiaticus* Astafurova & Proshchalykin, 2022 and *E.nudiventris* Bischoff, 1930. The hitherto unknown male of *E.mikhailovi* Astafurova & Proshchalykin, 2021 is described, and lectotypes are designated for *E.ruficornis* Morawitz, 1875 and *E.vinogradovi* Popov, 1952.

## ﻿Introduction

**Figure 1.**
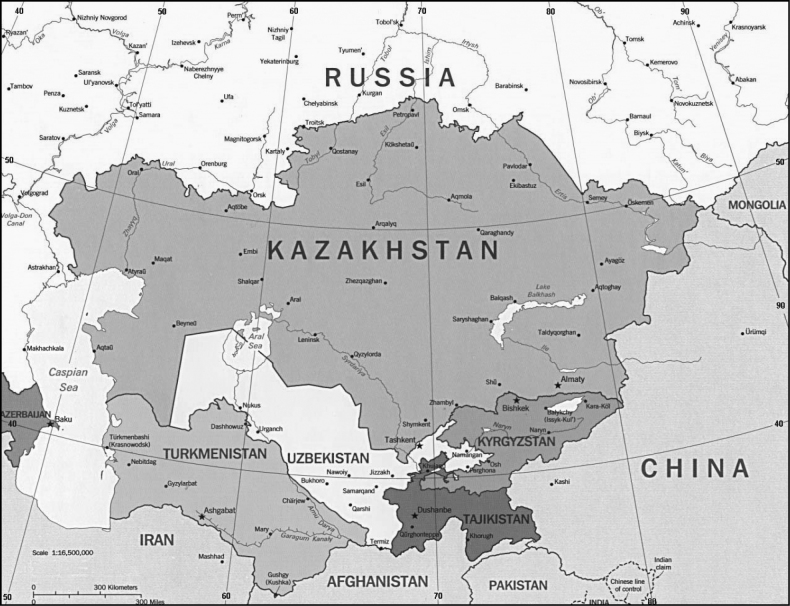
Map of Central Asia.

Central Asia as understood here covers the territories of Kazakhstan, Uzbekistan, Kyrgyzstan, Turkmenistan, and Tajikistan and has an area of about 4 million square kilometres, roughly half the size of Europe (Fig. 1). It comprises the arid to semi-arid regions of the Turanian Basin at its centre and the mountain ranges of the Tien Shan and Pamir in the east. Climatically and orographically the region is highly diverse, ranging from the lowland deserts of the Caspian Depression to the 7,495 m high Ismoil Somoni Peak in the Pamir. Aside from the Mediterranean Basin, Central Asia is the most important centre of bee diversity in the Palaearctic Region (Michener 1979). However, the bee fauna of Central Asia is underrecorded and, given the enormous size and ecological diversity of the area, the discovery of large numbers of undescribed species, including endemics, is highly likely (Kuhlmann and Proshchalykin 2013; Astafurova and Proshchalykin 2017; Dathe and Proshchalykin 2018; Astafurova et al. 2018; Wood 2023). Popov (1967) recorded about 1,200 bee species in 70 genera from this region and Ascher and Pickering (2023) list 1,360 species for this region, but the true number almost certainly is much higher.

The genus *Epeolus* Latreille, 1802 includes more 120 species spread across much of the globe; they occur throughout the Holarctic region, from the west coast of the United States and from Japan to Europe and North Africa (Michener 2007). A total of 59 species are known from the Americas (Onuferko 2018, 2019; Onuferko and Sheffield 2022) and about 50 from the Palaearctic region, of which 18 species are found in Europe (Bogusch and Hadrava 2018; Bogusch 2018, 2021; Astafurova and Proshchalykin 2021a, 2021b, 2021c, 2022a, 2022b). Unlike other Epeolini, all *Epeolus* species are so far known to be cleptoparasites of species of *Colletes* Latreille, 1802 (Colletidae).

*Epeolustransitorius* was the first species of the genus described from Central Asia (Eversmann 1852), and eight species have been described since from this area (Astafurova and Proshchalykin – four species; Morawitz – one species; Eversmann – one species; Radoszkowski – one species; Popov – one species), all of them still valid. Sixteen *Epeolus* species have been recorded from Central Asia so far (Astafurova and Proshchalykin 2021a, 2021c, 2022a, 2022b; Ascher and Pickering 2023). Based on a comprehensive study of specimens in various collections, we list here 20 species of *Epeolus* (Table 1) and describe as new two species from Central Asia for the first time.

**Table 1.** Records of Central Asian *Epeolus* species by countries.

*Epeolus* species	Kazakhstan	Uzbekistan	Kyrgyzstan	Turkmenistan	Tajikistan
*E.albus*	+	+		+	
*E.alpinus*	+				
*E.asiaticus*	+		+		
*E.cruciger*	+	+	+	+	
*E.gorodkovi*					+
*E.julliani*	+				
*E.kyzylkumicus*	+	+			+
*E.laticauda*	+	+		+	+
*E.mikhailovi*		+	+		+
*E.mongolicus*			+		
*E.nudiventris*	+	+	+	+	+
*E.pesenkoi*	+	+	+		
*E.productulus*	+	+			
*E.rasnitsyni*					+
*E.ruficornis*	+	+	+	+	+
*E.seraxensis*	+			+	+
*E.tarsalis*	+				
*E.transitorius*	+	+			+
*E.variegatus*	+				
*E.vinogradovi*				+	
***Total***:	**15**	**10**	**7**	**7**	**9**

A key to Central Asian *Epeolus* has not been included in this paper; it is forthcoming in a subsequent publication uniting this and the entire Palaearctic fauna due to their extensive sharing of species and the need for some additional work in these regions.

## ﻿Materials and methods

The results presented in this paper are based on 354 *Epeolus* specimens currently housed in the Zoological Institute, Russian Academy of Sciences (St. Petersburg, Russia, **ZISP**); Zoological Museum of the Moscow State University (Moscow, Russia, **ZMMU**); Federal Scientific Center of the East Asia Terrestrial Biodiversity, Far Eastern Branch of Russian Academy of Sciences (Vladivostok, Russia, **FSCV**); and Oberösterreichisches Landesmuseum, Biologiezentrum (Linz, Austria, **OLBL**).

Morphological terminology follows that of Michener (1944, 2007) and Engel (2001). The density of integumental punctures is described using the following formula: puncture diameter (in μm) / ratio of distance between punctures to average puncture diameter, e.g. 15–20 μm / 0.5–1.5.

Abbreviations **T** and **S** are used for metasomal tergum and metasomal sternum, respectively.

The species are listed alphabetically. We have used the following abbreviations for collectors: **MP** – M. Proshchalykin; **SB** – S. Belokobylskij; **VG** – V. Gurko; **VP** – V. Popov; **VR** – V. Rudolf.

Specimens were studied with an Olympus SZ51 stereomicroscope, and photographs were taken with a combination of stereomicroscope (Olympus SZX10) and digital camera (Olympus OM-D). Final images are stacked composites generated using Helicon Focus v. 7.7.4 Pro. All images were post-processed for contrast and brightness using Adobe Photoshop. New distributional records are noted with an asterisk (*).

## ﻿Taxonomy

### ﻿Genus Epeolus

Taxon classificationAnimaliaHymenopteraApidae

Latreille, 1802

C6691D0D-2E7E-57D3-AC2D-1E30172042FC

Epeolus Latreille, 1802: 427. Type species: Apisvariegata Linnaeus, 1758, monobasic.
Trophocleptria Holmberg, 1886: 233, 275. Type species: Trophocleptriavariolosa Holmberg, 1886, monobasic.
Epeolus (Diepeolus) Gribodo, 1894: 79. Type species: Epeolusgiannellii Gribodo, 1894, monobasic.
Epeolus (Monoepeolus) Gribodo, 1894: 80. Type species: Apisvariegata Linnaeus, monobasic.
Pyrrhomelecta Ashmead, 1899: 66. Type species: Epeolusglabratus Cresson, 1878, by original designation.
Argyroselenis Robertson, 1903: 284. Type species: Triepeolusminimus Robertson, 1902, by original designation.
Oxybiastes Mavromoustakis, 1954: 260. Type species: Oxybiastesbischoffi Mavromoustakis, 1954, by original destination.

### ﻿ Epeolus albus

Taxon classificationAnimaliaHymenopteraApidae

Astafurova & Proshchalykin sp. nov.

EA4C3DC7-F60E-567D-8C4B-5188A3972DCB

https://zoobank.org/494212D9-2ECE-454A-BFC7-7F0EBF23C191

Figs 2 , 3

#### Material examined.

***Holotype***: ♂, Uzbekistan: Kashkadarya Region, Yrta-Bulak, Sundukli, 16.V.2015, M. Proshchalykin, M. Mokrousov [ZISP]. ***Paratypes***: 1 ♂, the same label as in the holotype [ZISP]; 1 ♀, Bag-Absal, 50 km N of Buchara, 17.IX.1931, Zhelchovtsev [ZMMU]; Turkmenistan, 1 ♂, Kara-Bogaz, 40 km N of Kyzyl-Arvat, 31.V.1955, Odintzova [ZISP]; 1 ♀, Akhcha-Kuyma, 24.VI.1953, Steinberg [ZISP]; 1 ♀, 12 km SE of Tedzhen, 24.V.1964, A. Ponomareva [ZISP]; Kazakhstan, 2 ♂, Michailowskaja [=Taraz], coll. F. Morawitz [ZISP]; 1 ♂, Shelek, 30 km ENE of Habwüste, 43.41.24N 78.38.50E, 500 m, 2.VIII.2002, M. Kuhlmann [OLBL]; 1 ♀, Djulek, Syr-Darija, 24.VIII.1913, A. Gutbier [ZISP]; 1 ♀, 3 km NEE of Borandysu, 13.VI.2004, V. Kazenas [ZISP].

#### Diagnosis.

**Figure 2.**
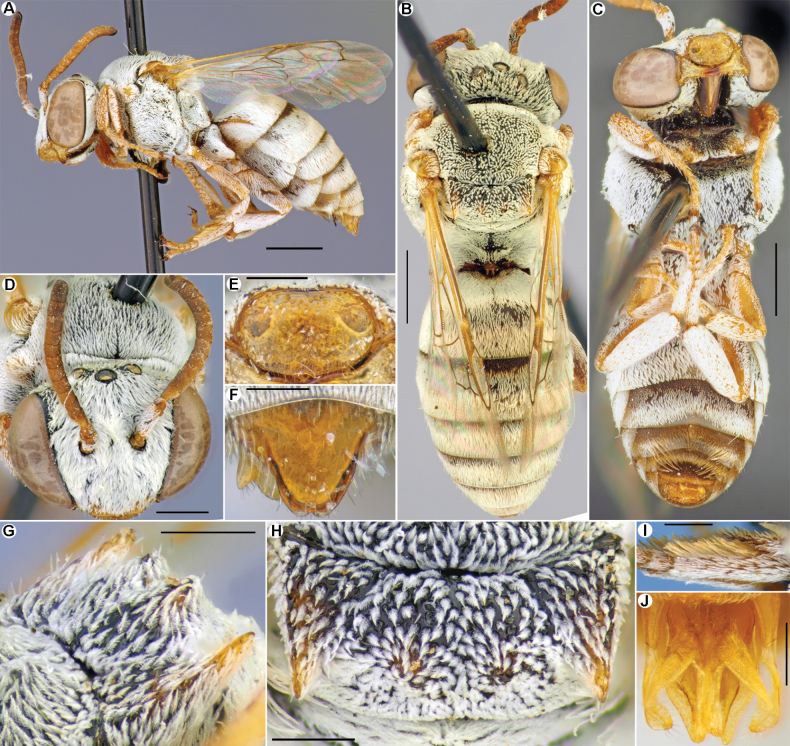
*Epeolusalbus* Astafurova & Proshchalykin, sp. nov., male, holotype (**A–I**), paratype (**J**) **A–C** habitus, lateral (**A**); dorsal (**B**) and ventral (**C**) views **D** head, frontal view **E** labrum, frontal view **F** pygidial plate, dorsal view **G, H** mesoscutellum, dorsolateral (**G**) and dorsal (**H**) views **I** hind basitarsus, lateral view **J** genitalia, dorsal view. Scale bars: 1.0 mm (**A–C**); 0.5 mm (**D**); 0.25 mm (**E–J**).

*Epeolusalbus* sp. nov. resembles *E.vinogradovi* Popov, 1952, *E.flavociliatus* Friese, 1899, *E.ruficornis* Morawitz, 1875, *E.subrufescens* Saunders, 1908, and *E.warnckei* Bogusch, 2018 in sharing the axillae with a pair of long, acute, curved teeth (free portion of axilla), reaching posterior margin of mesoscutellum or longer. Of them, the new species is most similar to *E.subrufescens* (Northern Africa, Middle East, and Turkey), with which it shares a pair of well-developed posteriorly directed processes (tubercles) between medial depression of the mesoscutellum (Fig. 2H) and has a male pygidial plate that is slightly bilobed apically (Fig. 2F) [vs weak developed mesoscutellar tubercles (Fig. 7D) and male pygidial plate rounded in *E.flavociliatus*, *E.warnckei*, and *E.ruficornis*)], but it can be separated from it by the well-developed pale pubescence of the metasomal terga that covers both the marginal zones (apical impressed area) and visible part of the discs (vs developed only as apical bands in *E.subrufescens*). In addition, *E.albus* has the labral teeth positioned more apically, while in *E.subrufescens* the labral teeth are positioned submedially.

In *E.vinogradovi* axillar teeth are more elongate and strongly curved; the mesoscutellum is with a pair of long, truncate teeth (Fig. 8D).

#### Description.

**Male (holotype).** Total body length 6.0 mm; forewing length (without tegula) 4.5 mm. Structure and sculpture: head (Fig. 2D) transverse, 1.3 times as wide as long. Labrum (Fig. 2E) 1.7 times as wide as long, rounded basally and laterally, apical margin almost straight without tooth; near apical margin with two poorly-developed teeth; integument shiny, densely punctate (10–15 μm / confluent–1). Flagellomeres about as long as wide. Axillae flat, apically with long tooth, extending over mesoscutellum (Fig. 2H). Mesoscutellum extending over propodeum; medially with pair small posteriorly directed processes (Fig. 2G). Pygidial plate (T7) dull, coarsely and densely punctate, 1.2 times as long as basal width, strongly narrowed toward apex; apical margin slightly bilobed (Fig. 2F). Sculpture of body not or hardly visible under pubescence; vertex, mesoscutum, and mesoscutellum densely punctate, interspaces shiny and smooth (15–25 μm / confluent–1.0); metasomal terga and sterna densely and finely punctate (10–15 μm / confluent–1). Genitalia as in Fig. 2J.

Integument coloration: head mostly black, but mandibles (excluding dark apex), labrum, clypeus on lower half, scape and antennae yellowish. Mesosoma mostly black; pronotal lobe, tegulae, axillar teeth, and legs (with pale spurs) yellowish; wings slightly infuscate, stigma light-brown, veins from yellow to brown. Metasomal terga black with pale and transparent marginal zones. Sterna brownish with yellow marginal zones. Pygidial plate yellow.

Pubescence: body with well-developed, snow-white tomentum of thick, plumose setae covering mostly of integument (Fig. 2A–C). Labrum almost glabrous, with few simple setae. Face and gena with dense tomentum of long, appressed setae obscuring underlying integument; vertex with sparser, short semi-erect setae. Mesosoma with dense, short, semi-erect setae obscured integument but little sparser than on mesoscutellum and axillae. Hind basitarsus bordered by a dense fringe of yellow long setae. Metasomal terga covered by white tomentum both on marginal zones and most of the visible part of discs (a little sparser on discs). S1–S3 with dense, white tomentum, especially on marginal zones of S2 and S3 (apical bands); S4 and S5 with long, golden setae.

**Figure 3.**
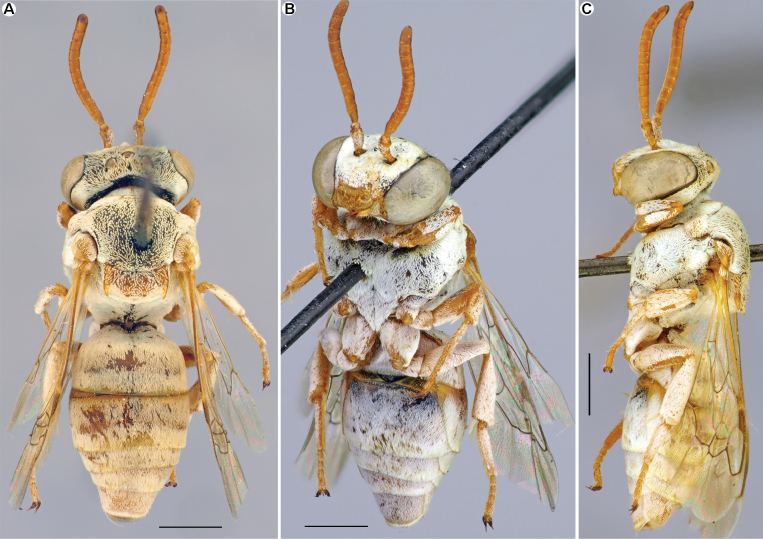
*Epeolusalbus* Astafurova & Proshchalykin, sp. nov., female, patatype **A–C** habitus, dorsal (**A**), ventral (**B**) and lateral (**C**) views. Scale bars: 1.0 mm.

**Female.** Structure, sculpture, and pubescence are similar to those of male (Fig. 3A–C). Antennae short, F1 1.25 times as long as wide; remaining flagellomeres slightly longer than wide. Sterna entirely covered by tomentum. Pseudopygidial area short, linear. Pygidial plate trapezoidal, apically truncate. Processes on sides of S6 normal, with short projections. S5 straight, as seen in lateral view. Integument coloration: reddish pattern of integument more developed than in male. Head mostly black, but mandibles (excluding dark apex), labrum, and clypeus yellowish; antennae reddish. Mesosoma black, except pronotal lobe, tegulae, axilla, mesoscutellum, and legs (with pale spurs) yellow to reddish; wings slightly infuscate, stigma light brown, veins from yellow to brown. Metasomal terga and sterna reddish with yellow marginal zones on S2–S4. Pygidial plate reddish.

#### Etymology.

The specific name “*albus*” (from Latin, meaning white) is associated with the extremely well-developed white pubescence of the body in the new species.

#### Distribution.

Desert areas in Kazakhstan, Uzbekistan, and Turkmenistan.

### ﻿ Epeolus alpinus

Taxon classificationAnimaliaHymenopteraApidae

Friese, 1893

6372B8D3-3156-55DF-81DA-2B1DE812392E

Epeolus alpinus Friese, 1893: 34, ♀, ♂ (type locality: Goeschenen, Switzerland).
Epeolus variegatus Thomson, 1872 (nom. praeocc., nec Linnaeus, 1758): 212, ♀ (type locality: unknown).
Epeolus glacialis Alfken, 1913: 36, nomen novum for E.variegatus Thomson, 1872.
Epeolus montanus Bischoff, 1930: 9, ♀, ♂ (type locality: Warnemünde, Germany).
Epeolus pilosus Bischoff, 1930: 9–10, ♀, ♂ (type locality: Rositten [=Rybachij], Kaliningrad Prov., Russia).
Epeolus alpinus Bischoff, 1930 (nom. praeocc., nec Friese, 1893): 9–10, ♀ (type locality: Saas, Switzerland).

#### Published data.

Astafurova and Proshchalykin 2022a: 308 (Kazakhstan).

#### Material examined.

Kazakhstan, 1 ♂, Chagan-Obo, Saur, Semipalatinsk distr., 13.VI.1910, leg. B. Karavayev [ZISP].

#### Distribution.

Kazakhstan; North Africa, Europe, Turkey, Iran, Russia (European part, Urals, Western and Eastern Siberia, Far East), Mongolia.

### ﻿ Epeolus asiaticus

Taxon classificationAnimaliaHymenopteraApidae

Astafurova & Proshchalykin, 2022

2CF3C860-F414-52FC-B50D-12CDFEA1C8D7

Epeolus asiaticus Astafurova & Proshchalykin, 2022a: 309, ♀, ♂ (type locality: Terkhin-Gol, Chulut and Khoit Rivers, Mongolia).

#### Published data.

No records in Central Asia.

#### Material examined.

Kazakhstan, 1 ♀, Semipalatinsk, coll. F. Morawitz (ZISP); 1 ♂, Aralsk, 23.V.1932, leg. Bening [ZISP]; 2 ♂♂, Alma-Ata, Medeo, 17.VII.1981, leg. Kocourek [OLBL]; 1 ♀, near Alma-Ata, 20.VII.1982, leg. Marikovskaya [ZISP]; Kyrgyzstan, 2 ♀♀, 2 ♂♂, Terskey Mts, Chong-Kyzylsu, 20.VII.1953, leg. D. Panfilov [ZMMU]; 1 ♂, Issyk-Kul, Teplokluchenka, 2000–2300 m, 18–20.VI.1995, leg. R. Rausch [OLBL]; 1 ♀, Bayduluu Range, Dolon Pass, 18.VII.1997, leg. Ovtshinnikov [OLBL]; 1 ♀, Alai Mts, Jkizjak, Kok-suu, VII.2000, leg. VG [OLBL]; 2 ♂♂, Firyuza, 7.VII.2001, Kocourek [OLBL].

#### Distribution.

*Kazakhstan, *Kyrgyzstan; Russia (Western and Eastern Siberia), Mongolia.

### ﻿ Epeolus cruciger

Taxon classificationAnimaliaHymenopteraApidae

(Panzer, 1799)

9E47608F-3705-58CD-B2E9-D7C3D494DFAC

Nomada crucigera Panzer, 1799: 20, ♂ (type locality: Austria).
Epeolus rufipes Thomson, 1870: 91, ♀ (type locality: S Sweden).
Epeolus similis Höppner, 1899: 355–356, ♀, ♂ (type locality: Freisenbüttel, Germany).
Epeolus cruciger var. elegans Müller, 1921: 168, ♀ (type locality: Arnswalde, Germany).
Epeolus cruciger var. rufiventris Müller, 1921: 168, ♀ (type locality: Arnswalde, Germany).
Epeolus marginatus Bischoff, 1930: 11, ♀, ♂ (type locality: Warnemünde, Germany).

#### Published data.

Morawitz 1880: 371 (Kazakhstan, as *E.rufipes*); Popov 1954: 365 (Kazakhstan); Astafurova and Proshchalykin 2021c: 11 (Kazakhstan); 2022a: 319 (Kazakhstan, Turkmenistan, Uzbekistan, Kyrgyzstan).

#### Material examined.

Kazakhstan, 1 ♀, Kyzylorda, Perovsk district, Kara-Uzyan, 15.IV.16, leg. N. Pulikovskaya [ZISP]; 1 ♀, Syr Darja River, Kazalinsk distr., 7.VI.1926, leg. Rukavishnikov [ZISP]; 1 ♂, Altay Mts, 10 km ENE of Nikitinka, Koktau Mts, 7.VIII.1983, leg. SB [ZISP]; 1 ♀, 25 km SSW of Zhansugirov, Aksu River, Zhungarskiy Alatau, 1600 m, 26.VII.1986, leg. Yu. Pesenko [ZISP]; Kyrgyzstan, 1 ♀, Ussyk-Kyl-Tal, Umg. Ortotokoj, 1700 m, 20.VII.1997, leg. V. Dolin [ZISP]; 1 ♀, 3 ♂♂, 30 km ESE of Rybachiy, Issyk-Kul Lake, 15.VII.1979, leg. Yu. Pesenko [ZISP].

#### Distribution.

Kazakhstan, Uzbekistan, Kyrgyzstan, Turkmenistan; Europe, Turkey, Syria, Iran, Russia (northeast to Magadan Prov.), Mongolia.

#### Remarks.

In Central Asia *Epeoluscruciger* is common only in northern Kazakhstan, but in the remaining Central Asian territories this species is rare and occurs mostly in the mountains. Here females have a mostly well-developed red pattern in the integument coloration and yellowish pubescence.

### ﻿ Epeolus gorodkovi

Taxon classificationAnimaliaHymenopteraApidae

Astafurova, 2022

63637CFD-F9A7-50C6-A212-8EB0A321CEEB

Epeolus gorodkovi Astafurova in Astafurova and Proshchalykin 2022a: 324, ♂, ♀ (type locality: Pamir Mts, Murgab River Valley, Tajikistan).

#### Published data.

Astafurova and Proshchalykin 2022a: 324 (Tajikistan).

#### Material examined.

No additional specimens examined.

#### Distribution.

Tajikistan; Afghanistan.

### ﻿ Epeolus julliani

Taxon classificationAnimaliaHymenopteraApidae

Pérez, 1884

80FDEC32-3BA1-5D71-8F17-F30AB508D1FB

Epeolus julliani Pérez, 1884: 318–322, ♀ (type locality: Marseille, France).

#### Published data.

Astafurova and Proshchalykin 2022b: 195 (Kazakhstan).

#### Material examined.

No additional specimens examined.

#### Distribution.

Kazakhstan; North Africa, Middle East, Europe, Caucasus, Russia (south of European part, southern Urals), Iran.

### ﻿ Epeolus kyzylkumicus

Taxon classificationAnimaliaHymenopteraApidae

Astafurova, 2022

3984EE30-593D-5D14-86D5-28F10C95719E

Epeolus kyzylkumicus Astafurova in Astafurova and Proshchalykin 2022b: 197, ♀, ♂ (type locality: 10 km SW of Arnasay, Kyzyl-kum, Uzbekistan).

#### Published data.

Astafurova and Proshchalykin 2022b: 197 (Kazakhstan, Uzbekistan, Tajikistan).

#### Material examined.

No additional specimens examined.

#### Distribution.

Kazakhstan, Uzbekistan, Tajikistan.

### ﻿ Epeolus laticauda

Taxon classificationAnimaliaHymenopteraApidae

Bischoff, 1930

9CE2D192-5DE4-5D4F-BB2B-4C3E3B1FCC51

Epeolus laticauda Bischoff, 1930: 13, ♂ (type locality: Mondy, Buryatia Republic, Russia).

#### Published data.

Popov 1935: 358; 1949: 695 (Tajikistan); 1967: 85 (Tajikistan, Uzbekistan); Astafurova and Proshchalykin 2021c: 11 (Tajikistan, Turkmenistan, Uzbekistan); 2022b: 201 (Kazakhstan, Tajikistan, Turkmenistan, Uzbekistan).

#### Material examined.

Tajikistan, 1 ♂, 15 km SW of Shaartuz, 2.VI.1982, leg. SB [ZISP].

#### Distribution.

Kazakhstan, Uzbekistan, Turkmenistan, Tajikistan; Russia (Eastern Siberia).

### ﻿ Epeolus mikhailovi

Taxon classificationAnimaliaHymenopteraApidae

Astafurova & Proshchalykin, 2021

547E66F8-E66A-52E0-9DB2-16365BF9843A

Fig. 4

Epeolus mikhailovi Astafurova & Proshchalykin, 2021a: 30, ♀ (type locality: Kyrgyzstan, Toguz-bulak).

#### Published data.

Astafurova and Proshchalykin 2021a: 30; 2021c:11 (Kyrgyzstan, Tajikistan).

#### Material examined.

Uzbekistan, 1 ♀, Samarkand, coll. F. Morawitz [ZISP]; Tajikistan, 1 ♂, Yagnob, 15.VI.[1870], [*E.transitorius* det. F. Morawitz] [ZISP]; 1 ♂, Zararavshan valley [Pasrut River, near Anzob], 24.VI.[1870] [*E.transitorius* det. F. Morawitz] [ZMMU]; 1 ♀, Pamir, Vanch, 30.VIII.1943, leg. Stakelberg [ZISP]; 3 ♀♀, 3 ♂♂, Pamir, Shavoz, Shakhadara River, 2800 m, 9.VII.1960, leg. L. Zimina [ZMMU]; 1 ♀, Khorog, 2600 m, 15.VI.1956, leg. Zhelokhovtsev [ZMMU]; 1 ♂, Pamir, Chil Dara, 12.VI., leg. N. Bogoyavlenskiy [ZISP]; 1 ♂, Pamir, 10 km SE of Ishkashim, 2600 m, 18.VII.1964, leg. Tanasiychuk [ZISP].

#### Description of hitherto unknown male.

**Figure 4.**
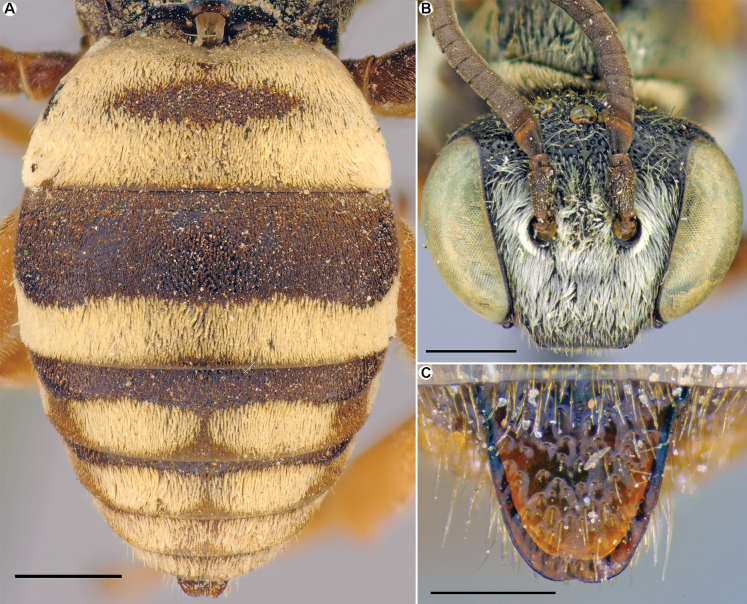
*Epeolusmikhailovi* Astafurova & Proshchalykin, 2021, male **A** metasoma, dorsal view **B** head, frontal view **C** pygidial plate. Scale bars: 1.0 mm (**A, B**); 0.25 mm (**C**).

Structure, sculpture, coloration and pubescence are similar to those of the female (see Astafurova and Proshchalykin 2021a: 30). Total body length 5.0–8.5 mm. Flagellomeres about as wide as long (Fig. 4B). Pygidial plate (T7) coarsely and densely punctate, 1.0–1.1 times as long as basal width, slightly narrowed toward apex; apical margin straight (Fig. 4C). Head (Fig. 4B) mostly black, but mandible reddish with red-brown apex and F1 reddish apically. Mesosoma mostly black; tegula, tibia, tarsi (and sometimes femora) reddish. Tergal discs black, marginal zones yellowish; pygidial plate black or reddish. Sterna brownish or reddish with yellowish marginal zones. Clypeus, paraocular and supraclypeal areas with dense (obscuring integument) tomentum; genal area with sparser and short setae. Frons with sparse short plumose and long simple setae. Ventral part of mesosoma, pronotum, mesepisternum (at least upper half), and metanotum laterally with whitish tomentum obscuring integument. Mesoscutum with developed tomentum on anterior half and peripherally. Tergal marginal zones (on T1–T4) with uninterrupted bands of tomentum (Fig. 4A); T1 with basal band widely connected with apical band laterally. S2–S3 with white bands of tomentum on marginal zones and S4 and S5 with golden long setae.

#### Distribution.

Mountains of *Uzbekistan, Kyrgyzstan, and Tajikistan.

### ﻿ Epeolus mongolicus

Taxon classificationAnimaliaHymenopteraApidae

Astafurova & Proshchalykin, 2021

49D3676E-B773-597D-88E1-C834DC91AF40

Epeolus mongolicus Astafurova & Proshchalykin, 2021b: 19, ♀ (type locality: 40 km SW of Uliastay, Zavkhan, Mongolia).

#### Published data.

Astafurova and Proshchalykin 2022a: 324 (Kyrgyzstan).

#### Material examined.

No additional specimens examined.

#### Distribution.

Kyrgyzstan; Mongolia, Russia (Eastern Siberia).

### ﻿ Epeolus nudiventris

Taxon classificationAnimaliaHymenopteraApidae

Bischoff, 1930

566CDC6B-137C-5D7D-B93A-A766C43D1C00

Epeolus nudiventris Bischoff, 1930: 14, ♀, ♂ (type locality: Mondy, Buryatia Republic, Russia).

#### Published data.

No records in Central Asia.

#### Material examined.

Kazakhstan, 2 ♂♂, Kazalinsk, 4.VI.1900, leg. S. Berg [ZMMU]; 1 ♀, Baigakum, Dzhulek, 21.VI.1907, leg. D. Glasunov [ZISP]; 2 ♂♂, Korgaly-Kul, Perovsk distr., 4.VIII.1911, leg. V. Kozhanchikov [ZISP]; 8 ♂♂, Baigakum, Dzhulek, Syr Darja, 14–19.VI.1912, leg. L. Wollmann [ZISP]; 4 ♂♂, Tartugay, 3–15.VI.1929, leg. A. Shestakov [ZISP]; 2 ♂♂, 50 km S of Balkhash, 26–27.VI.1992, leg. J. Halada [OLBL]; 1 ♂, Lepsi, 19–20.VI.1995, leg. J. Halada [OLBL]; 7 ♀♀, 3 ♂♂, Charyn valley, W Chundza, 650 m, 29–31.V.2001, leg. M. Hauser [OLBL]; 1 ♂, Dobyn Quella, saxaul steppe, 650 m, 30.VII.2002, leg. M. Kulhmann [OLBL]; 1 ♂, Charyn River, 12 km W of Chunzha, 11.VI.2004, leg. V. Kazenas [ZISP]; Uzbekistan, 1 ♀, 2 ♂♂, Farab, Buchara, 6.V.1917, leg. L. Wollmann [ZISP]; 1 ♀, Dautkul Lake, 55 km N of Nukus, 26.V.1972, leg. V. Zaytzev [ZISP]; 4 ♂♂, Mirishkor distr., Sundukli desert, 21–22.IX.2017, leg. MP [ZISP/FSCV]; 2 ♂♂, Mubarek, 20–21.IX.2017, leg. MP [ZISP]; 1 ♂, Yozyovon Sands, 17–18.IX.2022, leg. MP [ZISP]; Turkmenistan, 1 ♂, Dort-Kuyu, coll. F. Morawitz [*E.ruficornis* variet., Morawitz det.] [ZISP]; Kyrgyzstan, 1 ♀, 9 ♂♂, 30 km ESE of Rybachiy, Issyk-Kul Lake, 15.VII.1979, leg. Yu. Pesenko [ZISP]; Tajikistan, 1 ♂, Staraja Pristan, Vakhsh River, 11.IX.1948, leg. VP [ZISP].

#### Distribution.

*Kazakhstan, *Uzbekistan, *Kyrgyzstan, *Turkmenistan, *Tajikistan; Mongolia (Khovd), Russia (Eastern Siberia).

### ﻿ Epeolus pesenkoi

Taxon classificationAnimaliaHymenopteraApidae

Astafurova sp. nov.

28DA2300-FB32-58DC-AE23-39D36AAECCF4

https://zoobank.org/ADCC0377-64BC-41FB-AFF9-1A6395E569D2

Figs 5 , 6

#### Material examined.

***Holotype***: ♀, Kazakhstan: 25 km SSW of Dzhansurov [Zhansugirov], Aksu River, Zhungarskiy Alatau, 1600 m, 26.VII.1986, Yu. Pesenko [ZISP]. ***Paratypes***: 16 ♀, 8 ♂, the same label as in the holotype (ZISP).

#### Additional material.

Kyrgyzstan, 2 ♀♀, 3 ♂♂, Kungei-Alatau, 2200 m, 6–10.VII.1999, 8–9.VII. 2001, leg. Z. Klyuchko [ZISP]; 4 ♀♀, 2 ♂♂, E Terskei Mts, Arashan, 2000 m, 24.VIII.1999, leg. S. Zonstein, Makogonova [ZISP]; 2 ♀♀, Issyk-Kul Lake, Teplokljuchenka, 1800 m, VIII.2002, leg. VG [ZISP]; 3 ♀♀, Kirgizskiy Ridge, Malinovka, 1650 m, 10.V.2000, leg. VG [ZISP]; 3 ♀♀, Bayduluu Range, Dolon Pass, 18.VII.1997, leg. Ovtshinnikov [ZISP]; Kasakhstan, 1 ♀, near Alma-Ata, Kasha-Suu, 1650 m, VII.2002, leg. VG [ZISP]; 1 ♀, Talas Mts, 3 km W of Dzhabagly, 5.VIII.2000, leg. Makogonova [ZISP]; 1 ♀, Aksay, 35 km W of Alma-Aty, 16.VII.1981, leg. Kocourek [OLBL]; 1 ♀, 10 km E of Osinovka, 800 m, 19.VII.2002, leg. M. Kuhlmann [OLBL]; 1 ♀, Alma-Ata env., hills, 30.VI.1977, leg. Pulawskij [OLBL]; 1 ♂, Dzhungarskiy Alatau, 10 km NE of Tekeli Kora-Tal, 1000 m, 23.VII.2002, M. Kuhlmann [OLBL]; 1 ♂, near Alma-Ata, 12.VII.1982, leg. Marikovskaya [ZISP]; 1 ♂, Bugaz River, 35 km N of Ashat, 31.V.1972, leg. I. Kerzhner [ZISP]; Uzbekistan, 2 ♂♂, Charki, leg. Glasunov, coll. F. Morawitz [*E.transitorius* Morawitz det.] [ZISP].

#### Diagnosis.

This species belongs to the *E.variegatus* species group [*E.compar* Alfken, 1938, *E.eriwanensis* Bischoff, 1930, *E.intermedius* Pérez, 1884, *E.turcicus* Bogusch, 2018, *E.productulus* Bischoff, 1930, *E.productuloides* Bogusch, 2018, and *E.variegatus* (Linnaeus, 1758)] in sharing the presence of labral tubercles positioned close to the middle of labrum (as opposed to apically or subapically) and the curved (as opposed to straight) S5 of the female. From other species of the group, the new species can be distinguished by the uninterrupted albeit medially narrowed apical bands of the metasomal terga and usually bright yellow tomentum on the body of the female.

#### Description.

**Figure 5.**
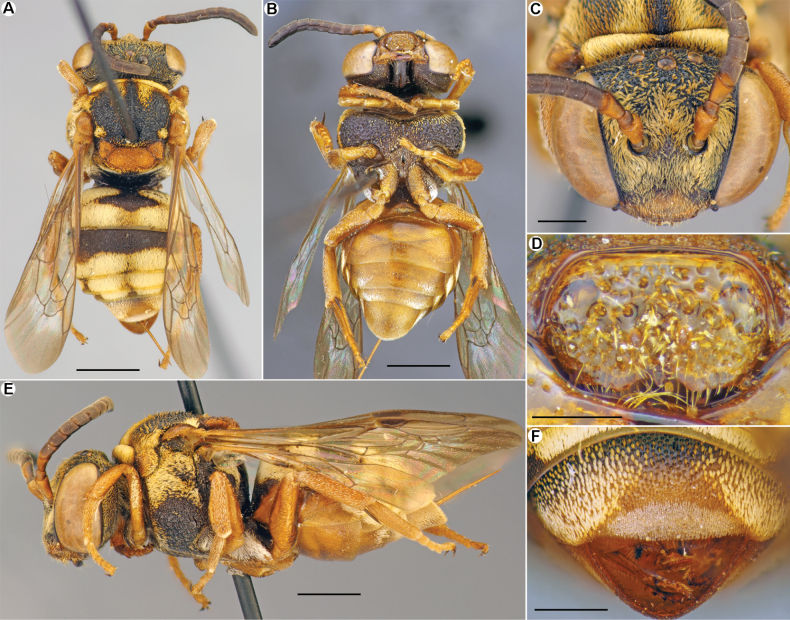
*Epeoluspesenkoi* Astafurova, sp. nov., female, holotype **A, B, E** habitus, dorsal (**A**), ventral (**B**) and lateral (**E**) views **C** head, frontal view **D** labrum, frontal view **F** T5, dorsal view. Scale bars: 1.0 mm (**A, B, E**); 0.5 mm (**C, F**); 0.25 mm (**D**).

**Female (holotype).** Total body length 7.0 mm; forewing length (without tegula) 5.5 mm. Structure and sculpture: head (Fig. 5C) transverse, 1.3 times as wide as long. Labrum (Fig. 5D) similar to those of *E.variegatus*; 1.6 times as wide as long, rounded basally and laterally, apical margin curved and with distinct medial tooth between two angulated lobes; submedially with two prominent teeth; integument shiny, coarsely and densely punctate (15–40 μm / confluent–0.5). Clypeus densely and finely punctate (10–15 μm / confluent–0.5). Frons with developed frontal keel. Frons and vertex coarsely and densely punctate, interspaces shiny (20–45 μm / confluent–1.0). F1 and F2 ca 1.3 times as long as wide; remaining flagellomeres ca as long as wide. Mesoscutum and mesoscutellum areolate-punctate (30–45 μm / confluent–1.5). Axilla short and flat, pointed apically, but without distinct tooth. Mesoscutellum with moderate deep longitudinal impression; posterior margin extending over propodeum. Mesepisternum finely areolate-punctate on upper half, lower part coarsely punctate with interspaces ca one puncture diameter. Propodeal triangle shagreened; rest posterior vertical surface of propodeum shiny and smooth. Metasomal terga finely punctate (10–15 μm / 0.5–2), interspaces shiny and smooth. Pseudopygidial area short (Fig. 5F). Pygidial plate trapezoidal, apically truncate. Processes on sides of S6 normal, with short projections. Sterna finely punctate with distinct interspaces (Fig. 5B). S5 curved as seen in lateral view.

Integument coloration: head mostly black, but mandibles (excluding dark apex), labrum, clypeus on lower half, scape and F1 amber (yellow-reddish). Mesosoma mostly black; pronotal lobe, tegulae, axillae, mesoscutellum, and legs (with dark spurs) amber; wings infuscate, stigma, and veins brown. Metasomal terga mostly black, but amber on posterior half of T5; marginal zones apically yellowish and transparent. Pygidial plate amber. Sterna (including marginal zones) amber.

Pubescence: pale tomentum mostly golden-yellow (Fig. 5A, E). Labrum with long simple setae near median teeth. Paraocular and supraclypeal areas with dense tomentum obscuring integument, clypeus with sparse pubescence. Upper half of frons with relatively long erect simple setae mixed with sparse appressed plumose setae. Vertex with sparse short and plumose setae. Gena with thick plumose setae on upper half (almost obscured integument) and with thin setae below. Pronotum dorsally with tomentum obscuring integument. Mesoscutum with paramedian strips of tomentum connected with lateral spots along pronotal lobe, pair small spots of tomentum posterolaterally and narrow strip along posterior margin (Fig. 5A). Mesepisternum with dense tomentum obscuring underlying integument on upper half, otherwise glabrous or with sparse setae. T1 with wide basal band of tomentum interrupted medially and connected with apical band laterally; marginal zones on T1–T4 with uninterrupted bands of tomentum. Setae on tergal discs dark brown; sparser than those comprising apical bands. Pseudopygidial area with silvery pubescence. Sterna with weak pubescence; S2 with simple short setae, S3–S5 with plumose short setae; marginal zones of T3–T4 with white tomentum laterally.

**Figure 6.**
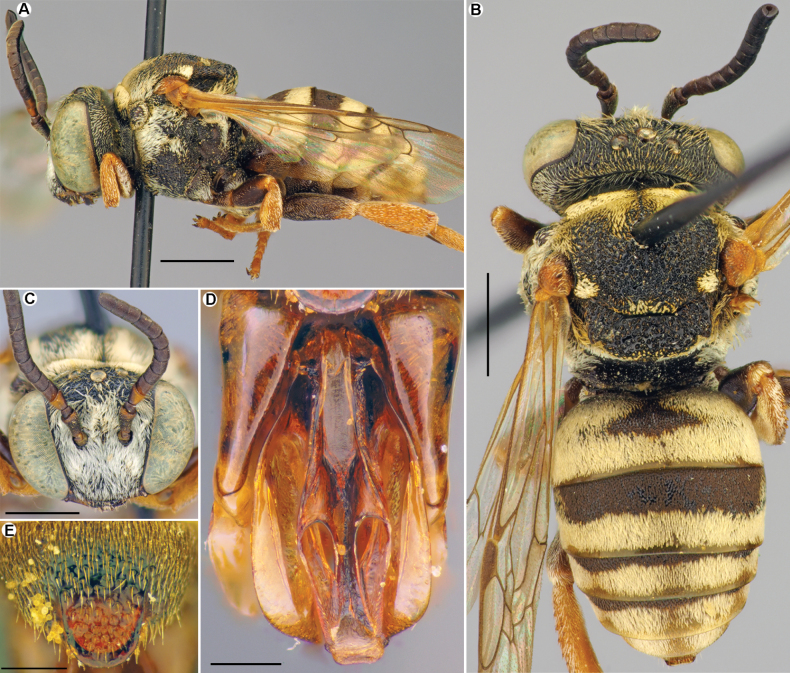
*Epeoluspesenkoi* Astafurova, sp. nov., male, paratype **A, B** habitus, lateral (**A**) and dorsal (**B**) views **C** head, frontal view **D** genitalia, dorsal view **E** pygydial plate. Scale bars: 1.0 mm (**A–C**); 0.25 mm (**D, E**).

**Male.** Structure, sculpture, coloration, and pubescence are similar to those of female (Fig. 6A–C). Flagellomeres about as long as wide. Pygidial plate (T7) reddish, shiny, coarsely and densely punctate, 1.1–1.2 times as long as basal width, slightly narrowed toward apex; apical margin curved (Fig. 6E). Clypeus, axillae, and mesoscutellum entirely black. Clypeus with dense tomentum obscuring integument. T1 basal band of tomentum uninterrupted. Marginal zones of S2 and S3 with dense uninterrupted white tomentum bands; S4 and S5 normal, with long, golden setae. Genitalia as in Fig. 6D.

#### Variability.

**Female.** Total body length 5.5–7.5 mm. Coloration of clypeus varies from entirely amber to dark brown/black along upper margin. The supraclypeal area is sometimes reddish on lower frontal keel. The metasomal terga are usually black (except amber on posterior half of T5) but may be reddish or brownish laterally along the marginal zones and on the posterior part of T1. The pubescence of the mesoscutum is usually as described in the holotype but sometimes has dense tomentum obscuring much of the anterior part instead of the usual distinct paramedian strips. Two specimens from the type locality have narrowly interrupted apical bands of tomentum. **Male.** Total body length 5.0–7.0 mm. The labrum varies from black to reddish. The basal band of tomentum on T1 is usually uninterrupted and is often almost merged with the apical band (except a small central area), rarely interrupted medially. Coloration of pale tomentum is usually less bright than in the female, from yellow to whitish.

#### Etymology.

The specific epithet is dedicated to Yuri Andreevich Pesenko (1944–2007), a renowned melittologist and, during his lifetime, one of the leading experts on the systematics of halictid bees.

#### Distribution.

Mountains of Kazakhstan, Uzbekistan, and Kyrgyzstan.

### ﻿ Epeolus productulus

Taxon classificationAnimaliaHymenopteraApidae

Bischoff, 1930

41F0DD9A-F076-5250-89B5-F138B99B2EFC

Epeolus productulus Bischoff, 1930: 4, ♂, ♀ (type locality: Sarepta, Volgograd Prov., Russia).

#### Published data.

Schwartz et al. 1999: 486 (Kazakhstan, Uzbekistan).

#### Material examined.

Kazakhstan, 3 ♀♀, Ber Tschogur, Mugodjar Mts, 3.VI.1910, coll. L. Wolmann [ZISP]; 1 ♀, Ryn-peski (desert), 12 km S of Urda, 3.VI.2001, leg. J. Miatleuski [OLBL]; Uzbekistan, 5 ♀♀, 1 ♂, Kurgan-Tyube, Fergana, 23.V.1938, leg. VP [ZISP]; 1 ♂, Dzhuma, Fergana, 23.V.1938 [ZISP].

#### Distribution.

Kazakhstan, Uzbekistan; Central and South Europe, Turkey, Syria, Ukraine, Russia (south of European part, Orenburg Prov.).

### ﻿ Epeolus rasnitsyni

Taxon classificationAnimaliaHymenopteraApidae

Astafurova & Proshchalykin, 2021

E9CAA75A-54D6-5B0F-B74B-00BAA0C2139D

Epeolus rasnitsyni Astafurova & Proshchalykin, 2021c: 11, ♂ (type locality: the mouth of the Shakhdara River, Tajikistan).

#### Published data.

Astafurova and Proshchalykin 2021c: 11 (Tajikistan).

#### Material examined.

No additional specimens examined.

#### Distribution.

Tajikistan (Gorno-Badakhshan Autonomous Region).

### ﻿ Epeolus ruficornis

Taxon classificationAnimaliaHymenopteraApidae

Morawitz, 1875

286120DF-E7BA-51DB-AA27-8A2F05573901

Fig. 7

**Figure 7.**
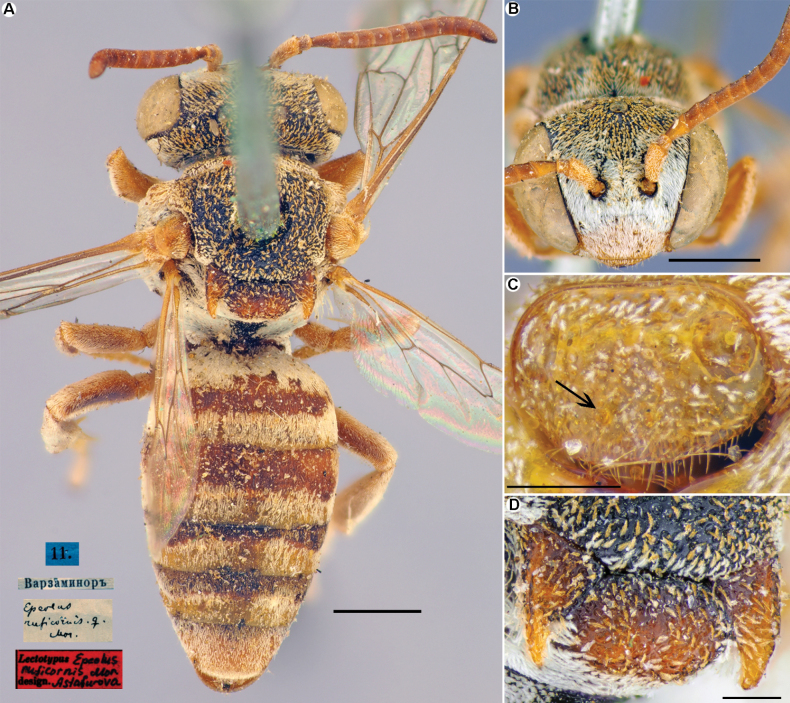
*Epeolusruficornis* Morawitz, 1875, female, lectotype **A** habitus, dorsal view and labels **B** head, frontal view **C** labrum, frontal view **D** mesoscutellum, dorsal view. Scale bars: 1.0 mm (**A, B**); 0.25 mm (**C, D**).

Epeolus ruficornis Morawitz, 1875: 144, ♀, ♂ (type locality: Ayni, Tajikistan). Lectotype (**designated here**): ♀, 11.VI.[1870] // Варзаминоръ [Varzaminor (= Ayni), Tajikistan // Epeolusruficornis Mor. [handwritten by F. Morawitz], [illustrated on Fig. 7], ZMMU, examined.

#### Published data.

Morawitz 1875: 144 (Tajikistan, Kyrgyzstan); 1894: 45 (Turkmenistan); Astafurova and Proshchalykin 2021c: 11 (Tajikistan, Turkmenistan).

#### Material examined.

Kazakhstan, 3 ♀♀, 3 ♂♂, Bayrakum near Dzhulek, 10.VI.1907, leg. L. Wolmann [ZISP]; 2 ♀♀, Dzhulek, Syr-Darja, 12.VI.1912, leg. L. Wolmann [ZISP]; 1 ♀, Kharkin, Ural River, 4.VIII.1951, leg. VR [ZISP]; 5 ♂♂, Koksengir, Zhana-Arka, 19.VII.1958, leg. A. Ponomareva, VR [ZISP]; 1 ♀, Kokshetau Mts, 2.VI.1959, leg. V. Tobias [ZISP]; 1 ♂, 50 km SW of Qyzylorda, 26.VI.1980, leg. D. Panfilov [ZMMU]; Kyrgyzstan, 2 ♂♂ (paralectotypes), Zeravhan River valley [near Aykul lake], 5.VIII.[1869], [ZMMU]; Turkmenistan, 1 ♂, Germab, coll. F. Morawitz [ZISP]; 1 ♂, Takhta-Bazar, 30.VI.1930, leg. VP [ZISP]; 6 ♀, 1 ♂, 30 km ESE of Rybachiy, Issyk-Kul Lake, 15.VII.1979, leg. Yu. Pesenko [ZISP]; Uzbekistan, 1 ♂, Khiva, Ravat, 26.V.1927, leg. V. Gussakovskij [ZISP]; 1 ♀, 2 ♂♂, Bukhara, Bag-Absal, 19.VII.1930, leg. V. Gussakovskij [ZISP]; 2 ♀♀, 1 ♂, Bag-Absal, 50 km N of Buchara, 17.IX.1931, leg. Zhelhovtzev [ZMMU]; 3 ♂♂, Dzhuma, 20.VIII.1957, leg. VP [ZISP]; 1 ♀, 10 km SW of Arnasay, Kyzyl-kum desert, 27.VIII.1979, leg. Yu. Pesenko [ZISP]; Tajikistan, 1 ♀, Varsaminor, 1892, leg. Glasunov [ZISP]; 5 ♀♀, 12 ♂♂, Kurgan-Tube, 30.VIII–5.IX.1948, leg. VP [ZISP]; 1 ♂, Staraya Pristan, Vakhsh, 12.IX.1948, leg. VR [ZISP]; 1 ♂, Vakhsh, 4–5.VI.1990, leg. J. Halada [OLBL]; 1 ♀, Khalton pr., Khodzhamulin Mts, 27.VI.2003, leg. Perepechayenko [ZISP].

#### Remarks.

The species is closest to *Epeoluswarnckei* Bogusch, 2018, but differs by having poorly developed labral teeth (Fig. 7C) which are positioned rather subapically (according to description of Bogusch (2018), in *E.warnckei* the labral teeth are positioned near the apex).

#### Distribution.

*Kazakhstan, *Uzbekistan, Kyrgyzstan, Turkmenistan, Tajikistan, Mongolia (Dornogovi, Khovd, Uvurkhangai), Azerbaijan, China (Xinjiang, Gansu).

### ﻿ Epeolus seraxensis

Taxon classificationAnimaliaHymenopteraApidae

Radoszkowski, 1893

2DCA8386-8E1C-5647-ACEC-B867C0A43F17

Epeolus transitorius var. seraxensis Radoszkowski, 1893: 54–55, ♀, ♂ (type locality: Serax, Turkmenistan).
Epeolus seraxensis : Bogusch 2021: 59. Upgraded to species rank.

#### Published data.

Bogusch 2021: 59 (Turkmenistan); Astafurova and Proshchalykin 2021c: 11 (Turkmenistan); 2022b: 207 (Kazakhstan, Tajikistan, Turkmenistan).

#### Material examined.

Kazakhstan, 3 ♀♀, Charyn River, 10 km NW of Chundzha, 22–23.VII.1988, leg. M. Volkovish [ZISP].

#### Distribution.

Kazakhstan, Turkmenistan, Tajikistan, Azerbaijan, Iran, Israel.

### ﻿ Epeolus tarsalis

Taxon classificationAnimaliaHymenopteraApidae

Morawitz, 1873

9880C410-BEDF-50A8-A12F-65B959300E04

Epeolus tarsalis Morawitz, 1873: 182–183, ♀, ♂ (type locality: Dagestan, Russia).
Epeolus praeustus Pérez, 1884: 324–326, ♀ (type locality: Pyrenees).
?Epeolussibiricus Radoszkowski, 1887: 295, ♀, ♂ (type locality: Vladivostok, Russia).
Epeolus rozenburgensis van Lith, 1949: 105–112, ♀ (type locality: the Netherlands).
Epeolus himukanus Hirashima, 1955: 40–41, ♂ (type locality: Kyushu, Japan).
Epeolus tarsalis ssp. tirolensis van Lith, 1956: 99, ♀ (type locality: Tirol, Austria).

#### Published data.

Astafurova and Proshchalykin 2021a: 32; 2021c: 11 (Kazakhstan).

#### Material examined.

No additional specimens examined.

#### Distribution.

Kazakhstan, Europe, Russia (European part, Eastern Siberia, Far East), Mongolia, Korea, Japan.

### ﻿ Epeolus transitorius

Taxon classificationAnimaliaHymenopteraApidae

Eversmann, 1852

BC31B8FE-A4ED-5C6C-85F7-93C0F8A8C42C

Epeolus transitorius Eversmann, 1852: 102, ♀ (type locality: Indersk District, Atyrau Province, Kazakhstan; ZISP).

#### Published data.

Popov 1951: 172 (Tajikistan); Le Divelec 2021: 16 (Kazakhstan); Astafurova and Proshchalykin 2021c: 11; 2022b: 209 (Kazakhstan, Uzbekistan, Tajikistan). The record of this species by Morawitz (1875: 144, 2 ♂) from Tajikistan belongs to *E.michailovi* Asatfurova & Proshchalykin, 2021.

#### Material examined.

No additional specimens examined.

#### Distribution.

Kazakhstan, Uzbekistan, Tajikistan; Greece, Georgia, Ukraine, Russia (southern European part, southern Ural, Western Siberia).

### ﻿ Epeolus variegatus

Taxon classificationAnimaliaHymenopteraApidae

(Linnaeus, 1758)

717137AF-5C10-51AC-8B78-DC0A38DD87E9

Apis variegata Linnaeus, 1758: 577, ♂ (type locality: Sweden).
Apis notata Christ, 1791: 188–189, ♀, ♂ (type locality: Germany).
Apis pulchella Christ, 1791: 194–195, ♀, ♂ (type locality: Germany).
Apis muscaria Christ, 1791: 195–196, ♀, ♂ (type locality: Germany).
Apis festiva Christ, 1791: 190–191, ♀, ♂ (type locality: Germany).
Epeolus pictus Nylander, 1848: 174–175, ♀, ♂ (type locality: Siberia, Russia).
Epeolus productus Thomson, 1870: 91, ♀, ♂ (type locality: Sweden).

#### Published data.

Popov 1934: 57 (northern Kazakhstan, as *E.productus* Thomson); Levchenko et al. 2017: 317 (Central Asia). The record from Tajikistan (22.VI – Anzob) (Morawitz 1875: 144) belongs to *E.transitorius*.

#### Material examined.

Kazakhstan, 1 ♀, Akmola, 6 km NE of Imkty-kol’ Lake, 21.VI.1957, leg. VR [ZISP]; 21 ♀♀, 59 ♂♂, Koksengir, S of Zhana-Arka, 31.VI–16.VIII.1959, leg. VR [ZISP]; 4 ♀♀, 4 ♂♂, Taldy-Manak River, 14.VIII.1959, leg. VR [ZISP]; 5 ♀♀, 3 ♂♂, Basaga-uzek River, 8.VI.1957, leg. VR [ZISP]; 2 ♀♀, 2 ♂♂, Yanvartzevo, 16–25.VI.1950, leg. VR [ZISP]; 5 ♀♀, 1 ♂, Atbasar, 3.IX.1936, leg. P. Rezvoy [ZISP]; 1 ♀, 3 ♂♂, 10 km N of Zharkol Lake, 17.VI.1957, leg. VR [ZISP]; 1 ♀, 1 ♂, Shabdar, 21.VI.1957, leg. VR [ZISP]; 1 ♀, 4 ♂♂, Kokshetau Mts, 12.VII.1958, leg. VR [ZISP]; 1 ♀, Borovoye, Kokchetav, 26.VII.1932, leg. VP [ZISP]; 1 ♀, near Uralsk, 5.VII.1908, leg. D. Borodin, V. Uvarov [ZISP].

#### Distribution.

Kazakhstan, North Africa, Europe, Russia (to Eastern Siberia), Turkey, Georgia, Kazakhstan, Iran, Pakistan, Mongolia.

#### Remarks.

In Central Asia *Epeolusvariegatus* is only known from northern Kazakhstan, where it is a common species. Reports of this species from other Central Asian countries are not confirmed by our examined material. In Russia, according to currently available data, the species is distributed east to the province of Irkutsk. A record from Yakutia by Davydova and Pesenko (2002) is a misidentification, which instead corresponds to *E.cruciger* and *E.alpinus*.

### ﻿ Epeolus vinogradovi

Taxon classificationAnimaliaHymenopteraApidae

Popov, 1952

0A3A0A54-7A5D-532D-AA16-7B6DDC5E0C50

Fig. 8

**Figure 8.**
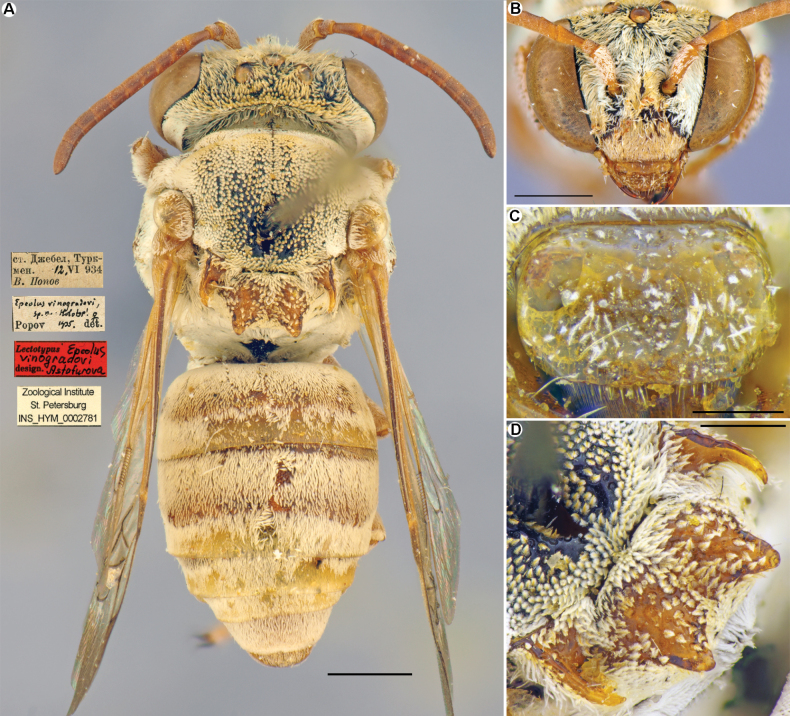
*Epeolusvinogradovi* Popov, 1952, female, lectotype **A** habitus, dorsal view and labels **B** head, frontal view **C** labrum, frontal view **D** mesoscutellum, dorsolateral view. Scale bars: 1.0 mm (**A, B**); 0.5 mm (**D**); 0.25 mm (**C**).

Epeolus vinogradovi Popov, 1952: 108, ♀, ♂ (type locality: Dzhebel, Turkmenistan). Lectotype (**designated here**): ♀, ст. Джебел, Туркмен. [Dzhebel, Turkmenistan], 12.VI.[1]934, В. Попов [V. Popov leg.] // к. Ф. Моравица // Epeolusvinogradovi sp.n. Holotype! ♀, Popov det. 1935 [illustrated on Fig. 8]; ZISP, examined.

#### Published data.

Popov 1952: 108 (Turkmenistan); Astafurova and Proshchalykin 2021c: 11 (Turkmenistan).

#### Material examined.

Turkmenistan, ♀ (paralectotype), Молла-кара, бл. Джебела, Туркм. [Molla-Kara, Dzhebel, Turkmenistan], 1.VII. [1]934, В. Попов [leg. V. Popov] // *Epeolusvinogradovi* sp.n. Paratype ♀, Popov det. 1935; 1 ♂ (paralectotype), Mulla Kara // *Epeolusspinosus* F. Morawitz [by Morawitz] // *Epeolusvinogradovi* sp.n. ♂, Popov det. 1935; 1 ♂ (paralectotype), Репетек [Repetek], 3.VI.27, Н. Умнов [leg. N. Umnov] // *Epeolusvinogradovi* sp.n. ***Paratype*** ♂, Popov det. 1935; 1 ♂, Uzun-Ada, 23.V.1896, leg. Varentzov; 2 ♂♂, Kara-Bogaz, 40 km N of Kyzyl-Arvat, 3.VII, 18.VI.1953, leg. Kryzhanovskiy; 1 ♀, Repetek, 8–13.VI.1976, leg. V. Kaplin.

#### Distribution.

Turkmenistan.

## ﻿Discussion

In total, 20 species are recorded from Central Asia, or, in other words, almost half of the Palaearctic fauna of the genus. For comparison, 18 species are known from Europe, 16 from the Middle East, 12 from North Africa, nine from Mongolia, 12 from Siberia and the Russian Far East, five from Japan, and only four from China.

The core of the *Epeolus* fauna of Central Asia is formed by seven endemic species (*E.albus*, *E.gorodkovi*, *E.kyzylkumicus*, *E.mikhailovi*, *E.pesenkoi*, *E.rasnitsyni*, and *E.vinogradovi*) and four species distributed from Central Asia to Mongolia and southern Siberia (*E.asiaticus*, *E.laticauda*, *E.mongolicus*, and *E.nudiventris*). Of them, *Epeoluslaticauda* is a fairly common species in desert biotopes. *Epeolusgorodkovi*, *E.mikhailovi*, and *E.rasnitsyni* occur mostly in the mountains.

Nine species are more widely distributed. *Epeolusjulliani*, *E.productulus*, and *E.transitorius* are widespread in the Palaearctic. *Epeolusruficornis* and *E.seraxensis* are distributed in arid territories of the Central Palaearctic, from the Middle East and Caucasus to Central Asia and Mongolia. Only four species recorded in Central Asia are widespread in the whole Palaearctic region (*E.alpinus*, *E.cruciger*, *E.tarsalis*, and *E.variegatus*), but in Central Asia they are rare (*E.alpinus*, *E.cruciger*, and *E.tarsalis*) or distributed only in northern part (*E.variegatus*).

## Supplementary Material

XML Treatment for Epeolus

XML Treatment for Epeolus albus

XML Treatment for Epeolus alpinus

XML Treatment for Epeolus asiaticus

XML Treatment for Epeolus cruciger

XML Treatment for Epeolus gorodkovi

XML Treatment for Epeolus julliani

XML Treatment for Epeolus kyzylkumicus

XML Treatment for Epeolus laticauda

XML Treatment for Epeolus mikhailovi

XML Treatment for Epeolus mongolicus

XML Treatment for Epeolus nudiventris

XML Treatment for Epeolus pesenkoi

XML Treatment for Epeolus productulus

XML Treatment for Epeolus rasnitsyni

XML Treatment for Epeolus ruficornis

XML Treatment for Epeolus seraxensis

XML Treatment for Epeolus tarsalis

XML Treatment for Epeolus transitorius

XML Treatment for Epeolus variegatus

XML Treatment for Epeolus vinogradovi

## ﻿Additional information

### Author ORCIDs

Yulia V. Astafurova https://orcid.org/0000-0003-0557-7792

Maxim Yu. Proshchalykin https://orcid.org/0000-0001-7870-8226

### Data availability

All of the data that support the findings of this study are available in the main text.
